# “Sometimes choices are not made, because we have ‘a’ choice, they’re made because they are ‘the’ choice”: Barriers to weight management for clients in rural general practice

**DOI:** 10.1186/s12875-022-01874-w

**Published:** 2022-10-25

**Authors:** Kimberley Norman, Lisette Burrows, Lynne Chepulis, Ross Lawrenson

**Affiliations:** University of Waikato, Gate 1, Knighton Road, Hillcrest, Hamilton, New Zealand and Waikato District Health Board, Pembroke Street, Private Bag 3200, 3240 Hamilton, New Zealand

**Keywords:** Obesity, Weight Management, Primary care, Rural Health, Qualitative, Patient perspective

## Abstract

**Background:**

Obesity is an international health issue which currently affects over 34% of New Zealand adults and leads to further physical and psychosocial health complications. People living in rural communities experience health inequities and have a high-risk of becoming obese. The aim of this study was to explore and identify barriers to effective weight management in rural Waikato general practice.

**Methods:**

Using semi-structured interviews, 16 rural Waikato participants shared their experiences with barriers to weight management. Interviews were transcribed and analysed using thematic analysis.

**Results:**

Four themes were identified: resource constraints, rural locality barriers, rural sociocultural norms barriers, and participants’ understanding the solutions needed to overcome their specific barriers to effective weight management. For these participants, finding a feasible weight management strategy was a challenging first step in their weight management journey. A programme that would ‘work’ meant one that was economically viable for low-income persons, accessible, even if living rurally with less resources, and did not cause harm or jeopardise their social connections within family or community.

**Conclusion:**

Overall, participants noted a lack of weight management strategy ‘choice’ because of income, isolation or accessibility of their rural location and/or the sociocultural norms of the community they lived in restricted options available to them. Future weight management initiatives may be better devised from within communities themselves and will need to be cognisant of the barriers specific to rural communities. Rural perspectives have much to offer in any such reconsideration of weight management initiatives.

## Introduction

Obesity is identified as a significant health concern affecting over 650 million people worldwide [1] with over 55% of the global rise in obesity reported to be from rural areas (from 1985 to 2017) [2]. The New Zealand (NZ) adult obesity rate is 34% [3] and is predominantly recognised as a risk factor for further health concerns by NZ’s Ministry of Health (MOH) [1, 4]. In the most recent NZ health survey (2002–2003) results demonstrated that rural females were more likely to be overweight or obese than urban females, while there was little difference between rural and urban males [5]. However, despite there being no updated rural obesity prevalence figure, it is likely that these rates have increased as the most recent reporting in the 2020/21 national health survey identifies that NZ overall obesity rate has increased from 31.2 to 34.3% in one year [3]. In NZ, the most at-risk populations for obesity are those living in socioeconomically deprived areas (1.6 times more likely to be obese) and those who identify as Indigenous Māori (51% obesity rate) or Pasifika (71%) [2, 3]. Rural towns are reported to experience more difficulties with access to primary healthcare than urban counterparts, with factors contributing to barriers including socioeconomic deprivation, rural geographical locality, transport, telecommunications and cost of healthcare [6], further increasing the risk of obesity development.

Contributors to obesity are recognised as more complex than an ‘excess calorie’ intake through food [7], with social determinants of health significantly influencing obesity development, especially for those living in rural areas [6, 8]. The obesogenic environments and political/ sociocultural systems in which people are born, live grow, work and age in can all influence obesity development [8–10]. The cost of fresh ‘healthy’ whole foods is out of financial reach for many low-income families, with processed (commonly high in carbohydrate, fat and sugar) foods readily available and pervasively marketed to those in lower socioeconomic areas [9, 11–13]. Populations living in rural areas experience less employment opportunities leading to high deprivation, limited local food stores or exercise facilities (driving prices up for importing goods and minimizing competitive markets), and less (or no) access to private car or public transport to access the resources they need for good health and effective weight management [6, 14]. Cultural norms have been demonstrated to dictate perceptions of ‘obesity’ with some cultures viewing excess weight as a positive phenomenon [15, 16], and therefore not warranting ‘treatment’ of weight management [17, 18]. However, acknowledging the myriad of individualised social determinants contributing to obesity, for those who want to lose weight or control their weight, weight management is an effective intervention and prevention strategy for obesity and its related comorbidities [19].

While acknowledging the significant role modern obesogenic environments and an individual’s choice to engage with weight management plays, one of the most effective ways to achieve weight management is through a combination of diet, exercise, and behavioural change conducted in culturally appropriate ways [19, 20]. This combination and balance of factors needs to be calibrated to the individual for suitability as no one diet suits all individuals. Many national health systems including the UK, Australia, Canada, America and NZ position primary care and general practice as best suited to deliver and offer clinical weight management guidelines to advise clinicians on best practice for treating and referring clients with obesity to specialists [19, 21–24]. However, weight management options are also available privately, through commercial avenues, or internet based information and sources for those who want to manage their weight themselves. Options within and outside the scope of general practice can include bariatric surgery, weight loss drugs/ medication, very low-calorie diets, meal replacement programmes, exercise programmes, commercial weight loss groups, telehealth or mobile app-based programs [19, 25–27]. However, the obesity rates are reportedly still rising in NZ, indicating that current weight management strategies in general practice are not effective, or potentially, not being used.

Through understanding what shapes rural experiences of weight management, valuable insights may be gained that can inform future obesity healthcare in the primary care space, enhance health outcomes, reduce obesity rates and increase quality of life.

The aim of this study was to explore and identify barriers to effective weight management in rural Waikato general practice from the client perspective.

## Method

### Participants

Participant criteria was > 25 years old, residing (or recently resided) in a rural Waikato geographical location and identified as someone with experience in weight management. While the individualised and subjective nature of obesity [28–30] is noted, for the purposes of this study all participants were currently, or have had, a BMI over > 30 (clinically obese) [31]. The definition of ‘rural’ has been a contested issue with some defining rural using empirical data and descriptive driven methods, and others using socio-cultural driven methods [32, 33]. However for the purposes of this health research, rural was defined as per the Geographical Classification of Health [34], which incorporates both data-driven and heuristic understandings of rural using a five-level rurality classification for health purposes [34]. Participants were recruited on a volunteer basis and purposeful sampling of males only was actioned towards the end as the sample was predominantly female [35]. A total of 16 participants were recruited from multiple rural locations to ensure data were not relegated to one locality which might have unique barriers, with demographic information listed in Table 1 below.

**Table 1:** Participant Demographic Data

Demographic	Participants (n)
Male	3
Female	13
Māori / NZ European and Māori	8
Non-Māori	8
Age 25–45 years	8
Age 46–70 years	8

### Data collection

Firstly, rural Waikato general practices and Māori health providers were contacted via phone and email and invited to participate. The general practitioners (GPs), nurses, and Māori healthcare professionals were asked to identify any of their clients that fit the criterion of this study. Once identified, they were asked to pass the researcher’s details to the client, or gain consent for their details to be passed to the researcher so they can be contacted. Secondly, snowballing strategy was utilised [35], whereby the researcher’s professional University and District Health Board networks and participants were asked to advise anyone they knew who fit the criteria to contact the researcher (5 participants were recruited this way). Once initial contact was made, the participant was given a copy of the information sheet and consent form for further details. All participant questions or concerns were answered and a suitable interview time and location was organised. Locations were chosen by the participant and included the researcher’s office, participant homes, cafes, local library, via skype, or their local general practice. All participants gave informed consent before any interviews commenced. A Māori cultural advisor was included throughout the research to ensure the Māori participant data were collected, analysed, and presented in a culturally appropriate manner [36].

### Procedure

Interviews were semi-structured to ensure that, although guided by a set of questions, participants were able to take the conversation in directions they wanted to throughout. At the beginning of each interview, the objective of the study was re-stated, and participants reminded they can end the interview at any time. All participants were offered space and time prior to the interview for culturally appropriate opening of meetings, such as prayer or karakia (Māori prayer). All interviews were guided by an interview guide which included open-ended questions such as: ‘could you please tell me about your experience with weight management?’, ‘could you please tell me about your experience with any barriers to weight related health engagements?’, ‘could you please tell me about your weight management experience living rurally?’. All participants were encouraged to speak about their experience for as long as they wanted to. Interviews lasted between 20 and 60 min and were audio recorded for later transcription. Participants were thanked for their participation and given a $30 voucher as a compensation for their time.

### Analysis

All interview data were transcribed verbatim for authenticity purposes and analysed using thematic analysis [37]. Each transcript was printed out, read, and re-read by the researcher with a view to facilitating immersion in the data. In the left-hand margin of each transcript, sections of conversation that reflected a ‘barrier’ to weight management in general practice were highlighted as this was main aim of this study. In the right-hand margin ideas that were significant to the participants’ discourse, including any obesity related barriers outside the general practice context were labelled permitting new or unexpected concepts to be identified and highlighted. These included the WHO defined [8] social determinants of health concepts (circumstances in which a person is born, lives and grows) such as poverty, housing, ethnicity, gender, and education. Each transcript was analysed in turn, and then comparatively re-analysed for any missing codes. All codes were listed, and redundant codes (that were found not to align with the aims of this study) were removed and double-up codes integrated. Whilst the ability to achieve data saturation is situated and subjective [38], this analysis found no new themes were being interpreted when revisiting the transcripts and reflecting on codes already identified [37]. The remaining codes were then grouped into overarching themes.

### Ethics approval

#### Ethical approval

was granted by the University of Waikato Human Research Ethics Committee reference HREC2020#38.

## Results

Four key themes emerged from the interviews relating to the interaction between living rurally and engaging with weight management strategies. These included resource constraints, rural locality, social norms of rural communities and the finding that participants had solutions to their own circumstances.

### Resource Constraints

Participants reported an awareness of effective health-enhancing weight management processes. Most participants shared an understanding that being a healthy weight involved eating healthy food (or less ‘junk’ food) and exercising more (or at all). If engaging with this behaviour, as one participant described it: “*of course you’re going to lose weight [it’s] basic maths” (Participant 01).*

Despite feeling reasonably comfortable in this knowledge, however, financial barriers shaped participants’ capacity to actually enact food-related or exercise-related strategies. Indeed, several participants framed the ability to engage with their health-related behaviours as a financial luxury. The cost of eating ‘healthy’ was notably difficult with *“the cost of food- it’s a lot cheaper to be unhealthy and to eat unhealthy food than it is to eat healthy food” (Participant 15).* Having the financial freedom to choose healthy options at the supermarket was unfeasible or unjustifiable for many participants as described by three participants:

*“Some days I have to decide between meat or vegetables” (Participant 05)*.

*“Potatoes are more cheaper than broccoli” (Participant 15)*.

*“A 2Litre coke is half the price of 2Litre of milk” (Participant 01).*

Several participants had heard about and were keen to embrace new dietary fads/prescriptions, yet understood these were beyond their means. As one woman declared, *“If you are going to do Keto, the biggest barrier is cost because the food- a lot of things you’re going to be eating is expensive- nuts, seeds and non- processed foods are expensive generally” (Participant 01).*

Several participants highlighted the ‘choice’ to engage with their desired health behaviours was also subject to time and availability. Spare time, personal time, or available time to spend on themselves was rare, as many other life responsibilities were prioritised before these health-enhancing actions such as *“kids and [work at school]” (Participant 06).* Low income, or lack of job security meant that the ‘choice’ of how to spend their own time was replaced with a ‘need’ for work and ensuring an income: *“I’m a freelancer so I don’t get anything like [sick days] so if I don’t work, I don’t get paid” (Participant 09)* or working to provide an income for their family *“I got a family I’ve got to work for” (Participant 10).*

Income was prioritised over health-enhancing activities. Participants working hours made accessing health facilities difficult, or impossible, due to their mostly inflexible opening hours, further removing a ‘choice’ from the individual. As one woman describes, the hydrotherapy pool can only be *“booked for certain times, and so if you work, you’re pretty much out” (Participant 09).*

To work around the lack of income or time barriers, personal sacrifices were often made by individuals to achieve their desired health-enhancing activities. However, these ‘pro-health’ sacrifices often generated potentially ‘unhealthy’ behaviours. For example, one man drunk straight olive oil as *“it was a more affordable way than buying a piece of salmon” (Participant 15)* and another reduced the amount of sleep she got to leave time for her morning walks *“I used to get up really early to do my walks in the morning so I could get it all done” (Participant 06).*

Community based exercise activities were sometimes available, however, access to these was again shaped by individuals’ financial situation. Low income precluded buying exercise equipment for personal use. As one participant described “*there’s no way I can buy all that sort of stuff myself” (Participant 05)* and that when *“you’re on a benefit, you can’t afford to things you can’t afford. For myself, it would have to come out of my food budget. There’s no leeway in it.”*

Health-enhancing options provided through general practice and the health system were out of range for many. An inability to afford clinically focused weight management options (such as medication and bariatric surgery) further restricted the ‘choices’ available to participants, as highlighted by two women: *“I didn’t want to go on Duramine again, it was extremely expensive” (Participant 14)* or bariatric surgery *“I can’t afford the surgery” (Participant 16).*

The capacity to make health enhancing ‘choices’ in rural communities, then was shaped by availability of money, time, and pressing responsibilities. As one participant highlighted: *“Sometimes choices are not made, because we have ‘a’ choice. They’re made because they are ‘the’ choice” (Participant 08).*

### Rural locality

Residing in a rural location was another barrier to participants’ ability to engage in health-enhancing activities. Rural locality meant isolation for some communities, which further limited ‘choices’ available to participants. In more out-of-the-way rural towns, access to common urban privileges, including internet service, public exercise facilities, or a variety of food store options was severely curtailed.

Rural locality was commonly compared with urban area ‘choices’ through the availability of supermarket choice (or at all) and weight management programme options *“[In the city] there were a lot more things to join” (Participant 07).* For both Māori and non-Māori participants, eating ‘healthy’ was *“challenging” (Participant 12)* when there is only a local dairy to shop at which stoked minimal fresh foods. The takeaway shop was positioned as a much more convenient and feasible option as one man described *“the price for the local shop down here, you can get a feed of fish and chips for about $7. As opposed to going to the supermarket” (Participant 11).*

The ‘out-of-town’ location made access to supermarkets difficult. Only those with the money, or those *“lucky”* enough to have the luxury of a *“car” (Participant 05 and Participant 06)* could travel to access them. For those who could not travel, the price of low-quality food was enhanced:

*“There is always a premium on prices here. Because the [shops] have to bring them in from wherever” (Participant 07).*

The inability to make frequent trips to the supermarket for fresh ‘healthy’ food also jeopardised the quality of the food participants had until their next shop.

*“That’s something to consider too, is the feasibility of getting the stuff fresh because if you only shop once a fortnight or some people only do once a month, they [have to] do a big shop and you don’t get all that other good stuff for the rest of the month. You might have it [good] for your first week, but then next three weeks you won’t have it” (Participant 06).*

Participants were also restricted in their exercise facility access due to the economic difficulties “*there’s still a cost to [getting to] them”* and rural locality challenges *“[its] an hour/ hour and a half to the nearest one” (Participant 05)*. Additionally, access to health-enhancing stores or facilities was subject to a participant’s available time, whereby work and childcare responsibilities often came first *“You can’t get it on the day you want, and then I work, and then straight after work I’ve got my kids” (Participant 10).*

### Social norms

Maintaining strong community social connections was important for these participants and abiding by rural social norms trumped weight management engagement for many participants. That is, social relationships were often regarded as more important than engaging with health-enhancing behaviours. For example, being helped by and helping fellow community members was a significant part of a rural lifestyle for many. One participant used her privilege of owning a car to help others less fortunate in the community and would pick up *“groceries for three or four people without cars” (Participant 05).* Another participant used their privilege of being able to go hunting and fishing and *“make up meat packs”* and “*deliver them to a lot of the Ko Matua (Māori Elders) and places of poverty” (Participant 06)* around their area.

Receiving help from the community was crucial for one participant to be able to engage in exercise activities in her home:

*“I do have a really old exer-cycle. But it’s piled up behind things at home, somebody is coming to help me to with that [and set up]. I’m lucky” (Participant 05).*

Rural communities were noted to have a different concept of ‘health’ compared with their urban counterparts, which was influenced by different sociocultural norms. Weight related health concept differences were also notably different than urban areas as one participant described *“in the rural communities, it’s okay to be a bit bigger” (Participant 14)* and another with a very *“different awareness about health” (Participant 07)* when compared with the urban capital city of Wellington.

Comparisons of clothing or physical appearance ‘expectations’ were also different as explained by one woman *“maybe 24 is not a normal size [in town], whereas in the small communities, it’s fine. Everyone is wearing gumboots and Swandry’s [farm clothes] anyway, you can’t see anything*” *(Participant 14).*

Rural social expectations also meant community participation was sometimes non-optional and further limited the individual’s health-related ‘choices’. The act of ‘dieting’ was viewed as not ‘the norm’ which could generate social tensions as one participant puts:

*“Smaller communities have a lot more community gatherings, which means a lot more food. So it’s almost expected that you participate and you enjoy everything, and you’re part of the community. Standing back there on a diet, or that kind of thing, gets looked at sideways” (Participant 14).*

Engaging with health-enhancing food options was difficult in social contexts for both Māori and non-Māori participants. Rejecting food was seen as ‘offensive’ and retaining social connections was prioritised over food choice, as one woman described:

*“For me the relationship with the person is more important, so this person has gone to the effort to cook it, so I’ll have to eat something. For me the social aspect is more important than wrecking a friendship over nothing” (Participant 01).*

Eating food you don’t want was a way to maintain the social connection that was vital for rural community living. One woman describes the ‘choice’ as being about maintaining a friendship or eating something that is not health-enhancing to her: *“you go to someone’s house and you don’t want the roast potatoes covered in butter” (Participant 01)* but it would be rude not to.

Additionally, specific cultural norms played an integral role in eating behaviours whereby rejecting food offered was unacceptable, further limiting the individual’s ‘choice’:

*“I guess for me also, being Māori and in a rural community is a huge issue. You would be completely disrespectful if you went to somebody’s house and they gave you food and you didn’t eat it” (Participant 14).*

For both Māori and non-Māori participants, ‘managing choices’ was difficult, because as one woman puts it: *“when you’re confined to a box, you can only choose what’s within it” (Participant 08).* This highlighted that those living in rural communities were aware of the multi-layered barriers of economic, rural locality and social norms they were subject to and how these influenced their ability to engage with their health-related behaviours.

### Solutions known already

The fourth theme was interesting and unexpected because when participants were asked about their rural experiences with barriers, participants gave solutions to their barriers unprompted. These ‘solutions’ were relative to each specific rural community as no two were the same, but suggestions covered themes such as exercise, diet, health literacy and the involvement of community. As one participant put it-

*“What helps Peter won’t help Paul. Especially when it comes to weight management” (Participant 05).*

For some this was free access to public exercise areas. One participant describes that a local park with exercise stations (such as a chin up bar, or lunges/ squats square, jumping jacks) is useful for her rural community and is *“more accessible than telling us to go to a gym, or go for a brisk walk” (Participant 06).*

Nutrition knowledge was positioned as important as well:

***“****[Knowing] where broccoli comes from versus where chicken nuggets come from. Like, what is chicken nuggets versus what is broccoli or chicken breast? I think that would change people, or maybe their mind, about what they’re putting into their body” (Participant 15).*

Culturally appropriate food or nutritional education specific to the community was also positioned as a solution that is needed as highlighted by one Māori participant:

*“We’ve gone away from healthy cultural eating. So working with community groups to encourage healthier eating like at Marae’s [Māori cultural meeting place] and stuff like that, or community festivals” (Participant 14).*

Enabling easier access to health professionals in rural areas was highlighted as a need for improving health also:

*“But also making it easier to have- having more community dieticians who are not just at the hospital, but in the medical centres and things like that, so you can easily get to see them” (Participant 09).*

## Discussion

Overall, the findings in this study highlighted a pervasive lack of ‘choice’ for rural participants when attempting or desiring to engage with weight management strategies which is relevant to rural areas worldwide. Participants were restricted in which health-enhancing behaviours they could engage with due to their economic income or resource constraints, isolation or accessibility of their rural location and the sociocultural norms of the community they lived in.

Previous research [8, 39, 40] has demonstrated that insufficient income contributes to poverty, housing insecurity, and mental health issues for populations across the world. Whitehead et al. [41] indicates that many rural Waikato clients travel significant distances to access general practice services, and Douthit et al. [42] highlights that those living in rural areas have isolation issues when accessing healthcare. It is little surprise then, that lack of a secure income and accessibility issues also shaped these rural participants’ capacity to engage in deliberate exercise and food-related behaviours.

As Kumanyika et al. [43] attests, sociocultural norms influence behaviours of communities and is evident in food and physical activity engagements in many cultures across the world. Sociocultural norms in relation to food behaviours in these rural areas included prioritizing social connections over food choice and the premise that rejecting food someone offers you was ‘offensive’, which risked jeopardising the social relationship. Further, Howard et al. [30] highlights that sociocultural factors influence the perception of weight status, whereby being ‘larger’ can be viewed as socially acceptable in rural areas and as such, does not pose a clinical health risk or align with the dominant obesity health discourse [28, 30, 44]. On the one hand, not being subject to the ‘thin ideal’ along with the negative effects of weight trends such as low self-esteem, body dissatisfaction and eating disorder development [45, 46] could be regarded as somewhat freeing from a body image/acceptability point of view. However, the normalisation of obese bodies in rural areas could be acting as a barrier to health-enhancing lifestyles whereby obesity, and consequently increased negative health risks, are misperceived as signs of wealth and beauty [47] therefore not needing ‘management’.

The positioning of solutions found in this research was unexpected and interesting because barriers were there before engagement in a weight management strategy. Previous reports have indicated many failed weight loss attempts are due to an individual’s lack of motivation to change [48, 49], however, participants in this study wanted to change and highlighted that there are barriers to weight management that are faced before a strategy is chosen or started. Their first choice of a strategy was usually unavailable to them for economic, sociocultural, or rural locality reasons. This indicates that there are significant difficulties faced before the ‘choice’ of a weight management plan is made, before a plan can be effective, or before general practice has even offered some form of healthcare. One interesting example was the lack of participants narrative around controlling of portion sizes, which is useful in weight management strategies, and is readily available advice through general practice or online at the MOH website [50] yet not utilised as a feasible strategy. For these participants, finding a feasible weight management strategy was a difficult first step in their weight management journey. For these rural participants, a programme that would ‘work’ meant one that was economically viable for low income, accessible even if living rurally with less resources, and not cause harm or jeopardise their social connections within their family or community.

Any attempt at intervention in rural areas, whether offered through general practice or not, needs to take into account suitability and feasibility for the lifestyles of the community members. While not an aim of this project, an unexpected finding was that participants expressed knowledge and awareness about what was needed in their community to overcome their barriers. While previous rural community research has indicated that people in rural areas are resilient, resourceful, and adaptive [51, 52] this research suggests that those living in rural areas also need support from their health system, environment, and social connections to overcome complex health risks such as obesity. Future weight management ‘interventions’ should use processes that align with community-based participatory research (CBPR) which works in a collaborative manner that strengthens, empowers and attends to social inequalities within a community [53]. As highlighted by the participants in this research, no rural weight management initiatives, or recommendations will be effective if the community does not have the money, public spaces, or time to enact them, regardless of whether interventions are within or outside the context of general practice. Any future healthcare efforts with rural communities across the world will need to address, and work within, the restrictive barrier limitations for effective health improvement outcomes, which CBPR has helped to achieve in the past [54]. Any interventions will need to be actioned on a community-by-community basis that address the unique local relevance of health problems and ecological barriers [53], which has been demonstrated to improve health outcomes in NZ and Māori specific communities [55–58].

As with any qualitative research, findings cannot be easily generalised, however, this research provides insights into the barriers faced by many communities across the large Waikato region which has not been explored before. This research looked at the barriers experienced by these rural communities, however, obesity is a complex health issue [1] and a deeper interpretivist analysis is needed for a more comprehensive understanding of barriers. Whilst the participants are from rural NZ areas, the findings are relevant to the international rural population who also face similar health inequity and disadvantages in their respective countries. This research acknowledges that themes could be different if designed from an indigenous worldview and that this study was not a Kaupapa Māori design, however, the barriers and themes were identified in both Māori and non-Māori narratives. Whilst this research focused on general practice clients in the Waikato region, minimal discourse in participants’ interviews was focused around this general practice context, suggesting further investigation should look into the significance and appropriateness of general practice for effective weight management outcomes. Future studies should investigate experiences of samples with more participants who identify as male and Pasifika participants. Community specific experiences should be explored with multiple/ all members of each community so a deeper, context specific understanding can be gained to mitigate barriers and improve health outcomes.

## Conclusion

This study identified four themes significant to rural general practice clients weight management experiences: resource constraints, rural locality barriers, social barriers, and that participants seemed to already understand the solutions needed to overcome their community specific barriers. Overall, participants had a pervasive lack of weight management strategy ‘choice’ because of their economic income or resource constraints, isolation or accessibility of their rural location and the sociocultural norms of the community they lived in. Future weight management initiatives need to be squarely located in communities where people need them and those who design them need a nuanced understanding of the particular barriers rural community clients face.

## Data Availability

The datasets generated and analysed during the current study are not publicly available due to the small rural geographical location where data were collected and the potential for identifying participants. The datasets are available from the corresponding author on reasonable request.

## Abbreviations

NZ: New Zealand
MOH: Ministry of Health
WHO: World Health Organisation
BMI: Body Mass Index
GP: General Practitioner
CBPR: Community-based Participatory Research
